# 2827. A mixed-methods analysis of provider-documented and patient-reported urinary tract infection symptoms among Veterans with neurogenic bladder

**DOI:** 10.1093/ofid/ofad500.2438

**Published:** 2023-11-27

**Authors:** Margaret A Fitzpatrick, Marissa Wirth, Pooja Solanki, Katie Suda, Stephen Burns, Frances Weaver, Eileen Collins, Nasia Safdar, Charlesnika T Evans

**Affiliations:** Rocky Mountain Regional VA Medical Center, Aurora, Colorado; Edward Hines Jr. VA Hospital, Hines, Illinois; Edward Hines, Jr. VA Hospital, Hines, Illinois; Pittsburgh VA Healthcare System, Pittsburgh, Pennsylvania; VA Puget Sound Health Care System, Seattle, Washington; Center of Innovation for Complex Chronic Healthcare, Hines, Illinois; University of Illinois Chicago, Chicago, Illinois; William S. Middleton VA Hospital, Madison, Wisconsin; Northwestern University and VA, Hines, Illinois

## Abstract

**Background:**

Urinary tract infections (UTI) are common in patients with neurogenic bladder (NB). Non-specific symptoms are frequent in this population, and may lead to inappropriate UTI diagnosis in patients who have asymptomatic bacteriuria. Characterization of patient-reported symptoms can inform interventions to promote appropriate UTI diagnosis.

**Figure:**
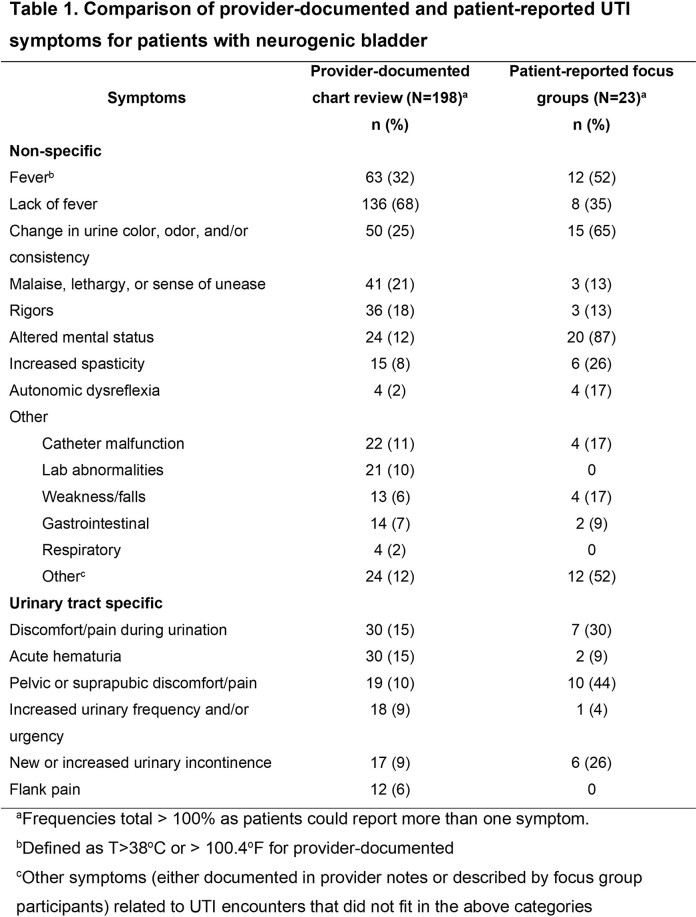

**Methods:**

Mixed methods study of adults with NB due to spinal cord injury/disorder (SCI/D), multiple sclerosis (MS), or Parkinson’s Disease (PD) and encounters with a UTI diagnosis (via ICD10). Provider-documented symptoms were collected from chart review of 198 encounters at 4 Veterans Affairs medical centers (VAMCs) between 2017-2018. Eleven virtual focus group discussions (FGDs) were conducted between May 2021 and May 2022 with patients from the chart review cohort with a UTI encounter in the prior year. Discussions were recorded, transcribed, and coded. Thematic analysis identified key concepts about symptoms and informed comparisons to provider-documented data from chart review.

**Results:**

Provider-documented (n=198 encounters; SCI/D 51%, MS 20%, PD 28%) and patient-reported (n=23 FGD participants; SCI/D 78%; MS 18.5%) symptoms are in Table 1. Similar to providers, most patients noted urine changes as UTI symptoms (“cloudy” and “smelly” urine). Only 12% of encounters had provider-documented altered mental status, but this was commonly described in FGDs (“brain fog”). Only 25% of encounters had provider-documented dysuria or pelvic pain, but this was also frequently noted in FGDs (“sitting on a stick”). Several patients felt providers ignored their symptoms, particularly when they did not have fever, which may affect provider documentation. Nearly all patients noted diminished quality of life from UTI symptoms, such as difficulty engaging in social activities and psychosocial impacts from worsened mobility and functioning.

**Conclusion:**

Both providers and patients attributed non-specific symptoms like urine changes to UTI, but there was discordance in recognition and reporting of other UTI symptoms. Optimizing care for patients with NB could involve patient and provider education on alternate sources of non-specific symptoms, interventions to improve communication about symptoms, and better assessment of quality of life impacts.

**Disclosures:**

**All Authors**: No reported disclosures

